# Artificial Intelligence in Religious Certification: A Comprehensive Review of Halal Digitalization, Compliance Integrity, and Future Directions

**DOI:** 10.1155/ijfo/3011089

**Published:** 2026-04-22

**Authors:** Mian N. Riaz, Fariha Irshad, Muhammad Bilal Haider

**Affiliations:** ^1^ Department of Food Science and Technology, Texas A&M University, College Station, Texas, USA, tamu.edu

**Keywords:** artificial intelligence, blockchain, food fraud detection, food traceability, halal certification, Internet of Things (IoT), predictive analytics

## Abstract

Global food supply chains continue to expand in scale and complexity, heightening risks of fraud, mislabeling, and cross‐contamination across certification systems. These vulnerabilities are particularly consequential for Halal certification, where ingredient permissibility, compliant slaughter practices, hygienic processing, and full traceability are religiously mandated. Artificial intelligence (AI), including machine learning, natural language processing, computer vision (CV), predictive modeling, and sensor‐enabled monitoring, offers transformative potential for strengthening the precision, transparency, and efficiency of Halal assurance. Current certification workflows often rely on manual document review, heterogeneous national standards, limited real‐time oversight, and susceptibility to fraudulent or unregulated Halal claims. AI‐enabled tools address these limitations by facilitating automated ingredient authentication through DNA‐based analysis, alcohol threshold detection, enzyme origin classification, and advanced label interpretation. In slaughter and processing environments, CV systems and IoT sensors enable continuous monitoring of animal handling, cut accuracy, bleeding efficiency, and postslaughter segregation, generating objective evidence of compliance. Pilot implementations indicate that integrating AI with blockchain‐based traceability and automated data pipelines can reduce certification timelines from weeks to days, improve anomaly detection to near‐perfect accuracy, and support dynamic, continuously verifiable compliance rather than periodic audits. Within this context, AI operates effectively under a hybrid governance model in which religious authorities retain interpretive and theological decision‐making while AI provides high‐resolution, tamper‐resistant documentation. Future advancements will depend on harmonized data standards, religious principle–aligned AI governance, strong ethical and cybersecurity safeguards, and wider regulatory acceptance across Islamic jurisdictions. Together, these efforts will enable next‐generation Halal certification while preserving religious integrity and sustaining consumer trust.

## 1. Introduction

Food certification provides formal, third‐party assurance that products meet defined standards for safety, quality, authenticity, and ethical production. These standards, established by governmental agencies, independent certification bodies, or religious authorities, verify that ingredients, processing methods, and handling practices comply with specified criteria such as microbiological safety, sustainability benchmarks, or dietary laws [1].

Among these systems, religious certifications, particularly Halal and Kosher, occupy a distinctive position because compliance reflects legally and spiritually mandated requirements rather than consumer preference. For Muslim and Jewish consumers, certification ensures that sourcing, slaughter, processing, packaging, and distribution adhere to scriptural guidelines designed to preserve both physical purity and spiritual integrity [2].

Globalization has significantly increased the complexity of food supply chains. Ingredients now traverse multiple countries and regulatory frameworks, often undergoing several stages of processing before reaching consumers. This fragmentation heightens vulnerabilities to mislabeling, adulteration, fraudulent claims, and accidental cross‐contamination. Even trace levels of prohibited substances, such as porcine‐derived gelatin, non‐Halal enzymes, or alcohol‐based carriers, can invalidate compliance, trigger recalls, erode consumer trust, and restrict market access [3]. These risks are magnified by the rapid growth of faith‐based food markets, including a Halal sector projected to exceed USD 2 trillion by 2030, supported by a Muslim population expected to reach 2.2 billion [4].

Traditional certification workflows, characterized by manual record inspection, periodic audits, and paper‐based traceability, struggle to keep pace with these increasingly interconnected and high‐volume supply networks. Ensuring accuracy and transparency across thousands of suppliers and intermediaries requires verification systems with analytical capacity beyond conventional methods [5]. Emerging evidence from food science and supply‐chain analytics demonstrates that artificial intelligence (AI) can markedly enhance detection accuracy, accelerate verification processes, and strengthen data integrity in complex agri‐food environments [6]. Machine learning (ML), computer vision (CV), natural language processing (NLP), and predictive modeling enable automated document interpretation, anomaly detection, and real‐time monitoring of processing conditions, while integration with blockchain and Internet of Things (IoT) sensors supports immutable traceability and continuous compliance surveillance [7, 8].

In the context of Halal certification, these technologies have the potential to support verification across all stages of the product lifecycle, from ingredient authentication and label analysis to monitoring of preslaughter animal welfare, slaughter compliance, and postslaughter segregation. However, because Halal determination is ultimately rooted in religious jurisprudence, AI functions as an evidence‐generation and risk‐screening tool rather than a decision‐making authority. Ensuring theological integrity requires a hybrid governance framework in which religious scholars maintain interpretive oversight while AI enhances the precision and scope of compliance assessment.

This review analyzes the potential of AI to modernize Halal certification systems. It first outlines the global certification landscape and current challenges, then examines the technological foundations and capabilities of AI relevant to Halal assurance, followed by applications, case studies, and forward‐looking considerations for developing scalable, ethically aligned, and trustworthy AI‐enabled certification frameworks.

## 2. Methodology

The objective of this review was to synthesize current knowledge on the application of AI in food certification systems, with a particular focus on Halal certification, compliance integrity, and digitalization. A structured literature search was conducted using Google Scholar, Web of Science, Scopus, and PubMed to identify relevant studies published between 2010 and 2025, while a limited number of earlier foundational references were included to provide contextual background. The search strategy employed combinations of keywords related to AI, ML, CV, NLP, Halal certification, food authentication, blockchain, IoT, and food traceability. Studies were selected based on their relevance to food certification or supply chain verification, their incorporation of AI or related digital technologies, and their applicability to Halal or comparable ethical and religious certification systems. Only peer‐reviewed publications and reputable sources with sufficient methodological rigor were included, while non‐English studies and those lacking technical or scientific relevance were excluded. The selected literature was analyzed using a qualitative, thematic approach, in which studies were categorized according to key domains such as certification challenges, AI technologies, supporting digital tools, and application areas. This analytical framework enabled the identification of major trends, research gaps, and emerging directions in AI‐enabled certification systems, ensuring transparency and consistency in the review process.

## 3. Food Certification Landscape

Food certification is a structured process of independent verification that ensures food products meet clearly defined benchmarks for safety, quality, authenticity, and ethical production. These benchmarks may be established by regulatory agencies, independent auditing bodies, or religious authorities and can range from basic food safety checks to complex sustainability and faith‐based requirements. Regardless of the category, certification serves as a public guarantee that a product has been produced, processed, and distributed according to recognized standards [9]. Increasingly, certification also operates as a risk‐management and traceability mechanism across fragmented global supply chains, where ingredients may pass through numerous intermediaries before final processing.

### 3.1. Categories of Food Certification

#### 3.1.1. Government Certifications

Governmental or intergovernmental agencies mandate or formally recognize certifications that prioritize public health, consumer protection, and trade harmonization. Examples include USDA Organic, which verifies production without synthetic fertilizers, genetic engineering, or prohibited pesticides [10]; the European Union Protected Designation of Origin (PDO), which protects the geographic authenticity of foods such as Parmigiano Reggiano cheese or Champagne [11]; and Codex Alimentarius standards jointly developed by the Food and Agriculture Organization (FAO) and the World Health Organization (WHO) [12]. Such certifications are often prerequisites for market entry and form the foundation of food safety, sanitation, and labeling accuracy, while also facilitating international trade. They further support regulatory harmonization by providing shared reference frameworks for producers and exporters operating within globally integrated supply networks.

#### 3.1.2. Third‐Party Certifications

Independent organizations and industry coalitions extend beyond regulatory compliance to reflect consumer‐driven values such as environmental sustainability, ethical sourcing, and authenticity [13]. Widely adopted examples include ISO 22000, an international food safety management standard; Fair Trade, which emphasizes equitable labor practices and sustainable farming; and the Non‐GMO Project Verified label, which confirms the absence of genetically modified ingredients through testing and supply chain audits [14]. While voluntary, these certifications hold significant influence, as they allow brands to communicate premium quality and responsible practices to increasingly value‐conscious consumers. Their adoption has expanded as companies respond to growing expectations for transparency, environmental accountability, and verifiable supply‐chain integrity.

#### 3.1.3. Religious Certifications

Religious food certifications address not only safety and quality but also spiritual and ethical compliance. Halal certification requires adherence to Islamic dietary laws, including permissible ingredients, ritual slaughter, hygienic production processes, and contamination‐free storage and distribution [15, 16]. Kosher certification, rooted in Jewish dietary law, stipulates acceptable animal species, bans mixing meat and dairy, and requires ritual slaughter practices [17]. These certifications are distinctive in that they combine technical oversight with theological authority, ensuring products meet both physical and spiritual benchmarks [18]. For Muslim and Jewish consumers, such labels are not only indicators of quality but also religious obligations, making accuracy and trust paramount [19]. Because they require specialized scriptural interpretation and oversight, religious certifications often involve more complex verification protocols than purely technical or regulatory systems.

### 3.2. Certification as a Trust‐Building Mechanism

Across all categories, certification acts as a mechanism of trust linking producers, regulators, and consumers [20]. It provides market access, enabling exporters to enter high‐value domestic and international markets where certifications such as Halal, Organic, or Fair Trade are prerequisites [21]. It enhances consumer confidence, allowing buyers to make informed choices on safety, sustainability, and ethical sourcing even when products originate from complex, multinational supply chains. Certification also strengthens risk management by reducing the likelihood of financial and reputational losses from contamination, fraud, or noncompliance [22, 23].

In today’s globalized food economy, where supply networks often span multiple continents, certification functions as a critical gateway to trade and as a signal of integrity. However, the scale and complexity of modern supply chains increasingly challenge traditional, manual certification models. Manual document review, periodic inspections, and paper‐based traceability systems are insufficient for real‐time monitoring and rapid verification in high‐volume, multicountry supply networks. This context underscores the urgency of adopting emerging technologies such as AI, which can reinforce accuracy, transparency, and efficiency in certification systems [24].

## 4. Halal Market Trends

Among all food certification categories, the Halal sector represents one of the most dynamic and rapidly expanding markets worldwide. Its growth reflects demographic trends, rising consumer incomes, and the mainstream adoption of Halal‐certified products by both Muslim and non‐Muslim consumers. The global Halal food market is projected to exceed USD 2 trillion by 2030, supported by sustained demographic expansion and evolving dietary preferences [25]. The worldwide Muslim population is expected to reach 2.2 billion, or approximately 26% of the global population, by 2030, providing a consistent demand base for Halal‐compliant food and beverages [26]. Rising disposable incomes in Muslim‐majority regions such as Southeast Asia, the Middle East, and parts of Africa have strengthened purchasing power, stimulating demand for premium and diversified Halal offerings [27]. Increased global mobility, cross‐cultural consumption patterns, and the proliferation of international retail chains have further accelerated the visibility and availability of Halal products across both Muslim and non‐Muslim markets. Urbanization and the emergence of younger, educated consumers further drive preferences for branded, processed, and convenience‐oriented Halal foods, accelerating the sector’s integration into global agri‐food trade [28]. This demographic shift also aligns Halal consumption with broader trends in health consciousness, ethical sourcing, and traceability, enhancing its appeal beyond religious communities.

### 4.1. Mainstream Adoption

Halal‐certified products are increasingly appealing to non‐Muslim consumers, who associate the certification with hygiene, traceability, animal welfare, and product integrity. This perception reflects a growing global preference for products verified through credible third‐party standards, especially in markets where consumers express heightened interest in clean‐label attributes and transparent supply chains. This trend has expanded Halal offerings into mainstream retail and foodservice sectors worldwide. Major supermarket chains in Europe and North America now stock Halal‐certified meat, snacks, and ready‐to‐eat meals for both Muslim and non‐Muslim consumers [29]. Multinational corporations, including Nestlé, Unilever, and Cargill, have invested in dedicated Halal product lines, underscoring the commercial potential of the sector as a driver of global competitiveness [30]. Such investments also signal a strategic shift in global food manufacturing, wherein Halal certification is increasingly viewed as a value‐adding mechanism for product differentiation and market expansion.

### 4.2. Key Halal Certification Bodies

Global Halal trade is underpinned by the activities of accredited certification authorities that establish standards, conduct audits, and issue certificates recognized across multiple markets. The Department of Islamic Development Malaysia (JAKIM) maintains internationally respected guidelines and collaborates with foreign certifiers to ensure mutual recognition [31]. In North America, the Islamic Food and Nutrition Council of America (IFANCA) is a major certifying body that audits facilities and verifies ingredients for multinational food companies [32]. The Indonesian Ulema Council (MUI) plays a critical role in Southeast Asia, certifying imports to Indonesia, one of the world’s largest Muslim consumer markets [33]. Other influential bodies, including the Halal Accreditation Council (Pakistan), Emirates Authority for Standardization and Metrology (UAE), and Halal Quality Control (Europe), contribute to increasingly interconnected regulatory networks that support cross‐border Halal trade. Similar agencies operate across Europe, the Middle East, and Africa, collectively shaping the institutional landscape of the global Halal industry [27]. Despite their shared objectives, variations in standards and jurisprudential interpretations across certifiers underscore the need for greater harmonization to support seamless international trade.

## 5. Challenges in Halal Certification

Despite its progress, Halal certification faces persistent challenges that threaten credibility and consumer trust. Multicountry supply chains, where ingredients and finished products pass through numerous intermediaries, increase the risk of mislabeling, adulteration, and cross‐contamination. The complexity of tracing ingredient origins is further intensified when processing steps occur in facilities that handle both Halal and non‐Halal materials, elevating the likelihood of unintentional contamination or ambiguous compliance status [9]. Variability in interpretation among Islamic schools of jurisprudence creates inconsistent requirements, particularly regarding animal stunning, processing aids, and allowable alcohol thresholds in flavorings. Exporters often require multiple certifications to satisfy differing national and religious standards. Fraudulent practices, including counterfeit certificates and unauthorized labels from unregulated entities, further complicate compliance and place legitimate producers at a disadvantage [34]. These inconsistencies create substantial regulatory friction and reduce confidence in cross‐border Halal labeling.

Halal certification thus represents both a major market opportunity and a regulatory challenge. Its continued expansion will depend on the ability of certifiers, governments, and industry stakeholders to strengthen credibility, harmonize standards, and improve transparency. The adoption of innovative digital solutions, such as blockchain, IoT, and AI, offers promising pathways to safeguard the integrity of Halal assurance while enabling the sector to meet the needs of an expanding global consumer base [35]. However, the effectiveness of these technologies depends on consistent data structures, clear verification rules, and robust governance frameworks that align technical outputs with religious requirements [36]. The major regulatory, operational, and technological challenges faced by current Halal certification systems are summarized in Table 1.

**TABLE 1:** Major challenges in Halal certification systems.

Certification stage/domain	AI technologies used	Functions and operations	Illustrative applications	Representative references
Ingredient authentication	Machine learning (SVM, random forest), NLP, deep learning (CNN/RNN)	Automated ingredient screening, origin tracing, detection of prohibited components, text mining of supplier records	Rapid flagging of non‐Halal enzymes, alcohol traces, or porcine DNA in formulations	[32, 37, 38]
Supply‐chain traceability and integrity	AI + blockchain, IoT sensors (RFID/QR), predictive analytics	Real‐time monitoring of temperature, humidity, segregation; tamper detection; predictive contamination risk	End‐to‐end Halal logistics verification from slaughter to export	[27, 39, 40]
Quality monitoring and process control	Computer vision, AI‐guided robotics, sensor fusion	Continuous observation of slaughter and hygiene, contamination detection, packaging verification	Computer‐vision monitoring of knife placement and bleeding efficiency	[41, 42]
Auditing and compliance management	NLP, machine learning, predictive analytics, remote viewing tools	Automated audit‐trail analysis, document verification, anomaly detection, virtual inspections	AI‐driven risk profiling for dynamic, continuous compliance	[5, 43, 44]
Consumer engagement and information	AI Apps, Chatbots, recommendation engines, image recognition	Barcode‐based Halal verification, personalized dietary guidance, counterfeit detection	Smartphone apps for Halal scanning and restaurant search	[45, 46]

The effectiveness of Halal certification systems hinges on their ability to uphold accuracy, transparency, and efficiency across increasingly complex global supply chains. These three pillars are critical for safeguarding religious integrity, strengthening consumer trust, and ensuring smooth trade between regions governed by diverse regulatory frameworks.

### 5.1. Accuracy

Every ingredient and processing step must be verified for Halal compliance. This includes confirming that raw materials, additives, and processing aids are free of prohibited substances, validating slaughter practices, and preventing cross‐contamination during production, storage, and transportation. Even a single undetected noncompliant input, such as porcine gelatin, lard‐based shortening, or alcohol‐based flavoring, can invalidate an entire production batch, trigger recalls, and erode brand reputation [47]. In modern supply chains, where manufacturers rely on multiple suppliers across different countries, verification becomes increasingly complex and error‐prone. The lack of standardized ingredient databases, inconsistent supplier documentation, and limited laboratory capacity in some regions further constrain accurate and timely verification.

### 5.2. Transparency

Certifiers, regulators, retailers, and consumers require access to clear, verifiable, and tamper‐proof records documenting a product’s journey from farm to shelf. Traditional systems, based on paper records and static audit reports, are slow to retrieve, vulnerable to manipulation, and inconsistent across jurisdictions and languages. The lack of real‐time visibility makes it difficult to trace contamination, detect fraud, or respond quickly to incidents, especially in cross‐border trade where products may pass through numerous intermediaries [35]. Incomplete documentation, undocumented subcontractors, and inconsistent reporting formats compound these challenges, creating gaps in traceability that undermine confidence in Halal claims. These issues contribute to consumer skepticism and highlight the urgent need for technologies that improve traceability and trust.

### 5.3. Efficiency

Conventional certification remains heavily dependent on manual inspections, multistage approvals, and repetitive document reviews. Manufacturers face long lead times for supplier verification, facility audits, and final certification approvals, delays that are magnified in export markets with differing Halal standards. Such inefficiencies disrupt production schedules, elevate warehousing costs, and increase the risk of missed shipping windows for perishable goods. For small and medium‐sized enterprises (SMEs), these delays can create disproportionate burdens, limiting market entry and competitiveness.

The absence of standardized digital platforms exacerbates the burden, as companies often need to resubmit overlapping documentation to multiple certifiers, resulting in costly duplication of effort. These operational challenges highlight structural vulnerabilities in the Halal assurance ecosystem. Without innovation, the industry risks undermining both credibility and competitiveness. Emerging technologies, particularly AI, blockchain, and IoT sensors, offer a pathway to overcome these bottlenecks by enhancing accuracy, enabling real‐time transparency, and streamlining certification workflows [48]. When properly governed, these tools can support continuous monitoring and high‐resolution verification while maintaining the theological oversight required for religious compliance. As explored in subsequent sections, integrating these technologies could allow the Halal industry to preserve religious integrity while advancing efficiency and reliability in global trade.

## 6. AI Technologies for Food Certification

The rapid digitalization of the food industry, often described under the framework of Industry 4.0, has introduced a new generation of technologies capable of analyzing complex datasets, detecting irregularities, and automating verification at unprecedented speed. At the center of this transformation is AI, which strengthens the integrity of food certification systems by reducing human error, increasing efficiency, and enabling real‐time oversight across globally dispersed supply chains [1]. By integrating with complementary technologies such as blockchain and the IoT, AI enables the development of fraud‐resistant, traceable, and scalable certification systems, addressing long‐standing gaps in accuracy, transparency, and efficiency. These capabilities are particularly relevant to Halal certification, where compliance requires continuous monitoring of ingredients, processing conditions, and slaughter practices across multi‐jurisdictional supply networks.

### 6.1. Definition and Core Capabilities of AI

AI encompasses a family of computational approaches designed to replicate or augment human cognitive functions such as learning, reasoning, and decision‐making. Within food certification, AI functions as a digital compliance assistant capable of processing large, multiformat datasets, including supplier documentation, ingredient specifications, inspection records, and audit reports, at a scale and speed far beyond manual review [43]. Importantly, AI is not a single tool but a suite of methods, each adaptable to specific certification challenges. When applied collectively, these methods create a multilayered verification ecosystem that can screen risks more comprehensively than traditional approaches.

Within Halal certification workflows, ML supports risk prediction, supplier classification, and anomaly detection based on historical compliance data. CV enables objective monitoring of slaughter practices, processing line segregation, and label authentication through real‐time image and video analysis [49, 50]. NLP automates the interpretation of multilingual ingredient lists, supplier declarations, and certification documents, identifying prohibited substances and aligning documentation with evolving Halal standards. Together, these techniques form an integrated analytical ecosystem that enhances accuracy, transparency, and efficiency across the certification process [51].

#### 6.1.1. ML

ML algorithms trained on historical data such as audit outcomes, supplier performance, and fraud incidents can predict risks and identify noncompliance patterns. These models detect correlations that may signal ingredient substitution, sanitation lapses, or fraudulent documentation long before they are apparent to human inspectors [52]. In Halal contexts, ML can also classify suppliers into risk tiers, enabling auditors to prioritize high‐risk facilities and ingredients for enhanced scrutiny.

#### 6.1.2. Deep Learning (DL)

Multilayered neural networks excel at uncovering subtle and nonlinear patterns in complex datasets. DL models can detect anomalies such as irregular batch codes, counterfeit certification marks, or unusual variations in supplier records, which may indicate intentional adulteration or unauthorized changes [53]. DL‐based imaging models can also be trained on thousands of validated slaughter images to evaluate cut accuracy, bleeding efficiency, and preslaughter handling practices.

#### 6.1.3. NLP

NLP systems enable automated analysis of unstructured, multilingual text. They can review supplier statements, translate documentation across languages, and identify prohibited ingredients, even when disguised under technical or trade synonyms. NLP also facilitates compliance monitoring by aligning supplier documents with evolving Halal standards [54]. This is particularly important because Halal‐relevant ingredients, such as enzymes, emulsifiers, and alcohol‐based carriers, often appear under obscure chemical or commercial names.

#### 6.1.4. CV

AI‐driven image and video analysis can authenticate certification labels, verify religious slaughter practices, and monitor production lines for contamination risks. Cameras equipped with CV algorithms can detect improper animal handling, identify prohibited ingredients, and flag counterfeit packaging in real time with consistency beyond human inspectors [42]. In slaughterhouses, CV can track knife placement, measure bleeding duration, assess animal movement postcut, and verify segregation between Halal and non‐Halal carcasses, providing high‐resolution evidence for certifiers.

In complex and dynamic environments such as animal handling and meat processing facilities, AI systems mitigate false positives and false negatives through ensemble modeling approaches. These systems combine convolutional neural networks (CNNs) for visual pattern recognition with temporal sequence models that analyze motion continuity and event duration rather than single‐frame observations [55]. Predictions are validated using confidence thresholds and temporal consistency checks, whereby alerts are generated only when deviations persist across multiple frames or time windows [56]. This approach reduces spurious detections caused by transient lighting changes, animal movement variability, or sensor noise, thereby improving reliability in real‐time Halal compliance monitoring [57].

#### 6.1.5. Predictive Analytics

Statistical and ML models can forecast emerging risks by integrating supplier history, geographic sourcing data, and production parameters. Such predictive capabilities enable proactive interventions, alerting certifiers to potential compliance breaches before they escalate into violations or recalls [58]. This transforms certification from a reactive model into a preventive, risk‐anticipation framework.

Together, these AI capabilities transform food certification from a reactive, document‐heavy process into a proactive, data‐driven risk management system. Instead of relying solely on periodic audits and manual inspections, certifiers and manufacturers can deploy AI to maintain continuous oversight, prioritize high‐risk suppliers, and safeguard compliance with safety, quality, and religious standards across complex international supply chains. For Halal assurance, this means providing verifiable, continuous monitoring across preslaughter, slaughter, and postslaughter stages, domains historically limited by manual inspection [59].

### 6.2. Supporting Digital Technologies

While AI provides the analytical engine for modern food certification, its effectiveness depends on a network of complementary digital technologies. Together, these systems capture high‐quality data, protect it from tampering, and maintain a continuous flow of verifiable information across global supply chains. This digital infrastructure forms the backbone that enables AI to function as a reliable compliance platform. Without secure, high‐resolution, and standardized data inputs, AI systems cannot produce accurate or interpretable compliance outputs.

#### 6.2.1. Blockchain

Blockchain operates as a decentralized, immutable ledger that records every transaction, ingredient movement, and certification update in a time‐stamped digital record. Each event in the production chain, from raw material sourcing to final packaging, is permanently logged and linked to a unique digital signature. When paired with AI, blockchain allows instant cross‐checking of supplier records, transport data, and certification histories, enabling the rapid detection of gaps or sudden anomalies that may signal fraud or adulteration [60]. Because records cannot be altered without network consensus, stakeholders, including certifiers, regulators, and consumers, gain a single, trusted source of truth that enhances the credibility of both Halal and other assurance systems [36]. Blockchain also facilitates cross‐border recognition of Halal certificates by providing a harmonized data trail accessible to multiple certifying authorities [61].

The blockchain architecture supports multiple stakeholders through permissioned nodes assigned to suppliers, certification bodies, regulators, retailers, and, where appropriate, consumers. Interoperability is achieved using standardized data schemas and application programming interfaces that allow different systems to exchange verified compliance information without compromising data integrity or access control. This structure enables cross‐border recognition while preserving jurisdictional autonomy [62].

#### 6.2.2. IoT Sensors

IoT devices embedded in slaughterhouses, processing facilities, transport containers, and storage units generate continuous, real‐time data streams on temperature, humidity, pH, sanitation cycles, and equipment usage. AI interprets these signals to confirm that production conditions align with Halal or Kosher protocols. For example, sensors can verify equipment sanitation before switching between non‐Halal and Halal production, detect temperature fluctuations that compromise food safety, or ensure that containers maintain the cold chain during global shipping. Real‐time alerts allow corrective action immediately, reducing dependence on periodic, after‐the‐fact audits [63]. Such systems increase operational confidence and provide certifiers with granular visibility into facility practices.

Sensor accuracy within AI‐enabled Halal certification systems is maintained through periodic calibration, redundancy across sensor types, and continuous data validation. ML models monitor incoming sensor streams for anomalies such as drift, discontinuities, or implausible values relative to historical baselines and adjacent sensors [64]. When inconsistencies are detected, the system flags the data for review, compensates using validated redundant inputs where available, or temporarily excludes faulty sensors from compliance assessment. This approach ensures reliable monitoring while preventing erroneous compliance conclusions caused by sensor malfunction [65].

#### 6.2.3. Spectroscopy and Rapid Testing Tools

Spectroscopic methods, such as near‐infrared (NIR) and Fourier‐transform infrared (FTIR) spectroscopy, provide chemical “fingerprints” of ingredients to detect minute variations in composition that indicate contamination or prohibited substances. Integrated with AI models trained in spectral libraries, these techniques achieve high sensitivity by recognizing subtle compositional patterns beyond the reach of traditional analysis. AI‐enhanced spectroscopy enables rapid, on‐site verification of high‐risk ingredients such as gelatin, emulsifiers, flavor carriers, and fat sources, reducing reliance on slow laboratory workflows [66].

#### 6.2.4. DNA‐Based Authentication

DNA testing offers a biological verification layer that is particularly valuable for complex processed foods where visual identification is impossible. Methods such as polymerase chain reaction (PCR) can detect trace amounts of nonpermissible animal DNA, providing high specificity in confirming ingredient identity. When integrated with AI, DNA‐based systems can automate result interpretation, classify outcomes, and generate standardized compliance reports for certification bodies, thereby improving both speed and reliability [67]. This is essential for verifying meat products, gelatin sources, enzyme origins, and other high‐risk materials in Halal certification.

By combining blockchain for secure recordkeeping, IoT sensors for continuous monitoring, spectroscopy for chemical verification, and DNA testing for species authentication, AI can cross‐reference diverse datasets to detect anomalies, predict risks, and recommend corrective actions. This multilayered verification ecosystem enables a shift from periodic, paper‐based inspections to continuous digital assurance, offering regulators, producers, and consumers unprecedented confidence in the integrity of certification systems [68]. For Halal assurance specifically, this integrated approach supports real‐time validation of compliance across slaughter practices, ingredient sourcing, and postproduction handling, ensuring that religious integrity is protected throughout the supply chain [59].

To address challenges associated with sample contamination or misidentification in DNA‐based analysis and enzyme‐origin classification, AI‐enabled verification systems rely on multilayer validation strategies. These include standardized sampling protocols, barcode‐linked chain‐of‐custody tracking, and cross‐validation using complementary analytical techniques such as spectroscopy and ingredient documentation analysis [68]. ML classifiers are trained to recognize atypical DNA degradation patterns common in highly processed foods and to flag low confidence or ambiguous results rather than generating definitive compliance outcomes. Confidence scoring thresholds ensure that isolated or uncertain detections do not trigger automatic noncompliance decisions. Final interpretations remain subject to confirmation by accredited laboratories and review by Halal certification authorities, preserving both analytical reliability and religious integrity [69].

## 7. Advantages of AI for Food Certification

Integrating AI with complementary digital technologies delivers transformative benefits across the three core pillars of effective food certification: accuracy, transparency, and efficiency. By shifting certification from periodic auditing to continuous digital assurance, AI enables more resilient, proactive, and scalable oversight that aligns with the demands of modern global supply chains. These advances are particularly relevant for Halal certification, where religious compliance requires flawless verification of ingredients, processing conditions, and supply chain integrity.

### 7.1. Accuracy

AI algorithms detect hidden risks with a level of precision that exceeds traditional manual inspections. ML models trained on historical compliance data can identify subtle patterns indicating contamination, ingredient substitution, or fraudulent documentation long before a human auditor would notice. DL models further enhance detection by capturing nonlinear abnormalities in batch codes, supplier behavior, or processing records that signify emerging risks [70]. CV systems analyze images and videos of production lines to identify visual cues of noncompliance such as improper slaughter techniques, cross‐contamination, or mislabeling. NLP scans multilingual supplier documents, translates technical terminology, and flags prohibited substances that might otherwise escape detection. When combined with spectroscopy and DNA‐based testing, these capabilities create multilayered verification pathways that minimize the likelihood of undetected errors and protect both consumer safety and brand integrity [71].

### 7.2. Transparency

Blockchain‐enabled AI allows regulators, certifiers, retailers, and consumers to access real‐time, tamper‐proof records of a product’s journey from farm to final packaging. Each transaction, ingredient movement, and certification update is recorded in an immutable ledger that can be instantly verified across borders [72]. AI amplifies this transparency by automatically scanning blockchain entries for anomalies, creating alerts when supplier histories are incomplete, when routing patterns deviate from expected pathways, or when certification records appear inconsistent. This combination of permanent digital records and intelligent anomaly detection reduces opportunities for fraud, strengthens consumer confidence, and supports international trade through simplified cross‐border verification [73].

Compared with paper‐based records or centralized databases, blockchain‐based systems provide immutable, time‐stamped records that cannot be retroactively altered, reducing opportunities for fraud and document manipulation. Decentralized verification enhances transparency by allowing authorized stakeholders to independently validate compliance data, while cryptographic security safeguards protect sensitive information. These features collectively strengthen accuracy, trust, and security within Halal certification workflows [74].

### 7.3. Efficiency

Automated document processing, predictive analytics, and real‐time monitoring sharply reduce the time and cost associated with audits and certification renewals. AI‐powered optical character recognition and NLP can extract and analyze supplier data within minutes, eliminating the need for manual review of lengthy records. Predictive analytics identify high‐risk suppliers or production batches and prioritize them for inspection, allowing certifiers to allocate resources more strategically. These efficiencies shorten certification timelines, reduce administrative burden, improve audit consistency, and facilitate faster product launches, particularly important for manufacturers serving multiple international markets with differing Halal requirements. As a result, companies experience lower warehousing expenses, fewer production delays, and improved agility in responding to market demand [47].

**FIGURE 1:**
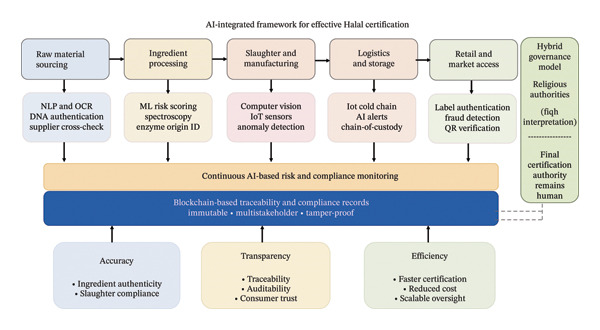
AI‐integrated workflow for effective Halal certification across the food supply chain.

These advantages are particularly significant for Halal certification, where religious requirements demand flawless ingredient verification, strict segregation during processing, and immediate detection of any noncompliant activity. By integrating AI with blockchain, IoT sensors, spectroscopy, and DNA authentication, certifiers can transition from infrequent, manual oversight to continuous digital supervision. The following section explores how these AI‐driven capabilities are already being applied, or are poised to be deployed, in Halal certification systems worldwide, demonstrating how technological innovation can safeguard religious integrity while enabling efficient and reliable global trade. Figure 1 illustrates an AI‐integrated framework for Halal certification across the food supply chain. AI tools, including NLP, ML, CV, spectroscopy, IoT sensing, and DNA‐based authentication, support continuous risk assessment from raw material sourcing to retail. Blockchain‐based traceability provides immutable compliance records, while a hybrid governance model ensures that final Halal certification decisions remain under the authority of religious experts. The framework enhances accuracy, transparency, and efficiency in Halal certification.

## 8. AI Applications in Halal Certification

The operational requirements of Halal certification, complete avoidance of prohibited inputs, strict segregation during processing, and precise documentation of every production step make it an ideal testbed for advanced digital tools. AI directly supports the three pillars of effective certification: accuracy, transparency, and efficiency. When combined with blockchain, IoT sensors, spectroscopy, and rapid DNA testing, AI enables certification bodies and manufacturers to move from slow, paper‐based audits to continuous, data‐driven compliance management. Recent advances also demonstrate the value of multimodal AI systems that integrate visual, chemical, biological, and textual evidence into unified decision‐support engines, enabling more robust and holistic Halal integrity verification across diverse production environments. Table 2 provides an overview of key AI tools currently applied across different stages of Halal certification, including ingredient verification, slaughter monitoring, fraud detection, and documentation validation.

**TABLE 2:** AI tools applied across Halal certification stages.

Category	Key benefits	Critical challenges	Shariʿah and ethical considerations	Representative references
Operational and economic	Faster certification, improved scalability, cost reduction, consistent outcomes	High setup cost, need for skilled AI + Halal experts, ROI uncertainty	Ensure equitable access (ʿadl); prevent hardship (darar); uphold fair trade (ihsan)	[7, 32]
Trust, transparency and integrity	Enhanced traceability, fraud prevention, data‐driven trust	Cybersecurity risks, inconsistent data quality, differing Halal standards	Uphold amanah (trust) and bayyinah (clarity) in data handling and AI outputs	[44, 75, 76]
Ethical and Shariʿah compliance	Evidence‐based ijtihad support; consistent interpretation of Halal criteria	“Black box” AI opacity, algorithmic bias, over‐reliance on automation	Maintain human oversight; AI must not replace Islamic Jurisprudence judgment; align with Maqasid al‐Shariʿah	[77, 78]
Regulatory and governance	Facilitates global standardization; supports international trade	Lack of AI‐Halal frameworks; liability and accountability issues	Develop Islamic‐ethics‐based AI governance; promote cross‐border standard harmonization	[79, 80]

The proposed AI‐enabled Halal certification framework extends beyond a linear workflow by integrating blockchain‐based traceability, predictive compliance modeling, and real‐time monitoring across all stages of the supply chain, from raw material sourcing to retail distribution. Rather than operating as a terminal verification step, these technologies function continuously, enabling fragmented and multi‐jurisdictional supply chains to be monitored dynamically [74].

### 8.1. Quality Control and Ingredient Verification

The foundation of any Halal certificate is assurance that every input and processing stage is free from non‐Halal substances. AI strengthens this foundation across multiple dimensions and provides rapid, highly precise detection and verification. In Halal contexts, this capability is particularly important given the religious prohibition of even trace amounts of haram substances and the need for evidence‐based verification aligned with religious principles.

#### 8.1.1. CV for Visual Inspection

High‐resolution cameras paired with deep‐learning algorithms monitor production lines in real time, capturing and analyzing visual data at a scale impossible for human inspectors. In slaughter facilities, CV can detect incomplete bleeding, improper knife placement, or the presence of non‐Halal carcasses before they enter processing. Advanced CV models can analyze blade angles, motion vectors, throat‐cut depth, time‐to‐collapse, and blood‐flow dynamics, parameters directly tied to Halal slaughter integrity. Pose estimation algorithms further assess animal orientation, preslaughter handling, stress behaviors, and neck positioning, allowing religious authorities to evaluate compliance with humane and approved religious practices.

In packaged foods, image analysis authenticates Halal logos, identifies counterfeit seals, and flags unauthorized use of certification marks, inspecting thousands of units per hour with consistent, objective assessments [81]. CV combined with generative AI can also detect manipulations in packaging artwork, microtypographic inconsistencies, and altered barcodes, key indicators of counterfeit or unauthorized Halal claims [41].

##### 8.1.1.1. AI‐Enhanced Spectroscopy

NIR and FTIR spectroscopy provide chemical fingerprints of ingredients by measuring the interaction of light with food components. AI algorithms trained on extensive spectral libraries distinguish Halal‐certified bovine gelatin from pork‐derived gelatin, detect traces of alcohol in flavorings, or verify that cooking oils contain only plant‐based fats. Deep neural networks enable sub‐band spectral feature extraction, making it possible to detect adulterants at parts‐per‐million levels. Multisensor fusion models now combine NIR/FTIR data with mass spectrometry, electronic nose (e‐nose), and e‐tongue profiles, creating comprehensive chemical authentication tools that support Halal ingredient verification even in ultra‐processed matrices [70]. By recognizing subtle spectral variations that may escape human analysts, AI increases sensitivity and reliability.

##### 8.1.1.2. IoT‐Enabled Process Monitoring

Sensors embedded in processing equipment continuously track temperature, humidity, pH, cleaning cycles, and equipment usage. AI interprets these data streams to confirm that equipment has been sanitized before switching from non‐Halal to Halal production and to identify unauthorized use of shared machinery. ML predictive maintenance models identify contamination‐prone “risk zones” such as valves, mixers, conveyors, and shared tanks. These models can forecast sanitation failures hours in advance, enabling corrective cleaning before contamination occurs. Real‐time alerts enable operators to correct deviations immediately, maintaining Halal integrity throughout every production shift [82]. IoT–AI integration also supports digital Halal audit trails by creating time‐stamped records of equipment conditions, sanitation intervals, and segregation protocols for religious certifiers [44].

##### 8.1.1.3. NLP for Documentation Review

Ingredient declarations often arrive in different languages and may use technical synonyms or trade names that complicate manual review. AI‐driven NLP automatically translates, interprets, and cross‐checks these lists against verified Halal ingredient databases. For example, the system can recognize that E441 or mono‐ and diglycerides may indicate pork gelatin or animal‐derived emulsifiers even when supplier documentation is incomplete or ambiguously worded [45]. Transformer‐based NLP models (e.g., BERT, RoBERTa) can identify hidden haram indicators, detect inconsistencies in supplier narratives, and extract compliance‐relevant metadata automatically. NLP systems can also map ingredient descriptions to evolving fatwa interpretations, helping ensure that documentation aligns with the most current religious rulings [83].

##### 8.1.1.4. DNA Testing With AI Integration

Genetic testing methods such as PCR detect trace amounts of prohibited animal DNA in highly processed foods where visual identification is impossible. AI accelerates the analysis of DNA results, classifies genetic material, and automatically generates standardized compliance reports. Deep‐learning sequence classifiers improve the detection of degraded DNA fragments, a common issue in processed foods, and can differentiate between closely related species such as bovine, buffalo, and porcine [84]. AI reduces the time from sample collection to regulatory action and provides a highly reliable safeguard against species substitution or hidden contamination. Some platforms now integrate DNA, spectroscopic, and image‐based evidence into unified dashboards, providing certifiers with multilayered verification snapshots for complex products [38].

##### 8.1.1.5. Collective Impact

Collectively, these tools transform quality control from periodic spot‐checking to continuous, predictive monitoring. By combining visual inspection, chemical analysis, sensor‐based process monitoring, automated documentation review, and genetic testing, AI‐enabled systems sharply reduce the probability that non‐Halal ingredients or practices enter the supply chain and provide certifiers with a robust foundation for issuing and maintaining Halal certification. This multimodal surveillance framework represents a major shift from reactive audits to real‐time compliance assurance.

#### 8.1.2. Automated Compliance Checks

Traditional Halal certification depends on manual cross‐referencing of supplier certificates, production records, and audit reports—a process that is time‐consuming and vulnerable to human error. AI shifts this approach from reactive inspection to automated, proactive verification by continuously monitoring documentation, production data, and supplier behavior.

AI rule‐based systems can encode Islamic jurisprudence and fiqh principles to ensure machine decisions remain aligned with religious interpretations, enabling a hybrid model in which AI supports, not replaces, religious authority. Fiqh refers to the systematic understanding and interpretation of Islamic law derived from the Qur’an and Sunnah to guide practical religious rulings in specific contexts.

Variations in Halal interpretation across Islamic schools of jurisprudence and geographic regions are addressed through a hybrid governance architecture. AI systems operate as decision support tools configured with rule‐based layers that reflect region‐specific Halal standards, fatwa interpretations, and certification body requirements. These rule sets can be selectively activated depending on jurisdiction or certifier, allowing technical consistency while respecting theological diversity. Importantly, AI‐generated outputs serve as structured evidence rather than final rulings, ensuring that accredited religious scholars retain ultimate authority over Halal determinations in context‐sensitive or disputed cases [79].

#### 8.1.3. Document Extraction and Verification

Optical character recognition combined with ML rapidly extracts key information such as ingredient names, supplier identification numbers, and expiration dates from complex multipage documents. The data are then compared to approved Halal ingredient registries or official supplier lists [37]. Advanced OCR also detects formatting anomalies, altered metadata, and manipulation signatures, which are common in falsified documents.

#### 8.1.4. Certificate Validation

AI systems match certificate numbers, issuer details, and digital signatures against official certification databases in real time. Expired, duplicated, or counterfeit certificates can be flagged instantly. Metadata forensics, such as timestamp verification, file lineage tracking, and signature cryptography, provide another layer of authentication that strengthens cross‐border trust.

This automated validation is particularly valuable for exporters that must maintain multiple certifications across different countries [85].

#### 8.1.5. Predictive Risk Assessment

By analyzing historical compliance data, AI identifies high‐risk suppliers, ingredients, or geographic regions with a history of noncompliance or fraudulent practices. ML models weigh inspection results, shipment irregularities, and production fluctuations. Risk scores allow certifiers to prioritize audits based on predicted likelihood of violation, enabling smarter resource allocation and strategic surveillance [86].

#### 8.1.6. Real‐Time Production Monitoring

With IoT integration, AI detects deviations such as unexpected temperature changes, improper cleaning cycles, or unauthorized ingredient substitutions. Alerts are sent immediately to plant managers before a noncompliant batch leaves the facility [41].

Centralized AI dashboards consolidate sensor outputs, risk scores, document verification results, and real‐time video analytics into a single interface accessible to certifiers, enabling continuous digital auditing [59].

By integrating these capabilities into a single digital platform, AI converts compliance from a periodic audit process into a continuous surveillance system critical for Halal systems where absolute accuracy is required.

### 8.2. Fraud Detection and Fake Certificate Identification

Fraudulent certificates and counterfeit labels undermine consumer trust, distort market competition, and create costly trade barriers for legitimate producers. AI provides complementary defenses that detect fraudulent activity faster and more accurately than manual inspection, reducing opportunities for deception within the certification process. Fraud in Halal certification often exploits weak documentation systems, inconsistent recordkeeping, and decentralized supply chains; thus, AI’s ability to fuse visual, textual, metadata, and blockchain‐derived signals provides a highly resilient multilayer defense.

#### 8.2.1. Image and Logo Recognition

Deep‐learning models analyze the placement, resolution, and microfeatures of official seals, holograms, and certification marks to detect forgery or unauthorized reproduction. Subtle irregularities such as small shifts in logo alignment, inconsistent font patterns, or pixel‐level distortions, often invisible to the human eye, can be flagged automatically on product packaging, shipping labels, or digital certificates to ensure every visual element matches the official standard [72]. Modern CV systems also compare scanned labels against authenticated digital templates stored in certifier databases. CNNs can identify adversarial forgeries, color spectrum anomalies, and manipulated printing textures. Generative AI detectors can reveal synthetic alterations or “deepfake packaging” increasingly used in counterfeit goods. These systems can screen thousands of packages per hour, enabling real‐time fraud surveillance at ports, warehouses, and retail outlets [87].

#### 8.2.2. Anomaly Detection in Supplier Behavior

ML algorithms monitor supplier documentation and identify patterns that deviate from normal operations. Sudden changes in formatting, inconsistent batch numbers, abnormal submission frequencies, or unexplained alterations in supplier histories can signal attempts to bypass certification requirements. By learning the normal rhythm of supplier activity, AI flags suspicious behavior in real time and alerts certifiers for further investigation [48].

Advanced anomaly detection models (e.g., LSTM‐based time‐series predictors) can monitor longitudinal behavioral patterns, detecting subtle irregularities such as repeated last‐minute documentation uploads, missing batch traceability links, or unusual production‐volume spikes [88]. AI can also analyze multisite supplier networks and identify coordinated fraud behaviors that would be impossible to detect manually.

#### 8.2.3. Metadata and Digital Signature Analysis

AI inspects the metadata of electronic documents, including creation dates, issuer identities, and encryption signatures, to verify authenticity. Discrepancies such as mismatched timestamps, missing security certificates, or inconsistent file histories provide early warning of counterfeit records. Automated analysis enables rapid validation of large volumes of digital files with greater confidence than manual review [70]. Metadata forensics can detect hidden document edits, cloned certificates, reused signatures, and altered PDF structures. AI systems cross‐reference encryption hashes with recognized certifying bodies to confirm whether a certificate originates from an accredited source. ML‐based tamper detection also identifies suspicious file genealogy, such as documents saved through third‐party unverified software or produced using image scans of forged originals [61].

#### 8.2.4. Blockchain Integration

When paired with blockchain, every transaction from ingredient procurement to final packaging is recorded in an immutable chain of custody. AI can scan these records for gaps or inconsistencies, such as a product appearing in a market without a corresponding export entry or a supplier change lacking proper documentation, creating a powerful defense against document tampering and unauthorized product substitution [43]. AI‐driven blockchain analytics (e.g., graph algorithms) can map supplier relationships, identify unusual linkages, and detect “ghost nodes” or fake intermediaries inserted to hide non‐Halal inputs. Outlier detection can flag impossible logistics sequences, such as transport durations shorter than feasible or supply volumes exceeding known production capacity [60]. Blockchain–AI synergy ensures that counterfeit certificates cannot be injected into the supply chain without detection.

#### 8.2.5. Integrated Multilayer Defense System

By combining image analysis, behavioral monitoring, metadata verification, and blockchain‐based traceability, AI establishes a multilayered defense system that protects consumers from mislabeled products, safeguards the reputation of legitimate certifiers, and supports transparent global trade by ensuring that only authentic Halal‐certified goods reach the market.

This integrated system forms the core of “Holistic Halal Fraud Intelligence,” in which simultaneous cross‐validation across modalities (visual, textual, digital, and transactional) creates a tamper‐resistant, highly adaptive fraud‐detection network. AI thus transforms Halal certification from a vulnerable paperwork‐based process to a technologically fortified assurance framework capable of detecting sophisticated fraud attempts across global supply chains.

### 8.3. Operational Efficiency and Accessibility of AI in Halal Certification

Halal certification is often criticized for being costly and time‐intensive, especially for SMEs seeking entry into competitive export markets. AI addresses these challenges by automating routine tasks, organizing complex documentation, and directing limited auditing resources to areas of highest risk [89]. AI‐powered systems handle repetitive tasks such as document collection, data extraction, and cross‐referencing of supplier records. Processes that once required several weeks of manual review can now be completed in days, enabling faster product launches and reducing the risk of shipment delays caused by pending certification. These efficiencies are amplified when AI operates within integrated digital ecosystems that merge IoT sensors, blockchain platforms, and cloud‐based audit management systems, enabling seamless data flow and eliminating redundant administrative burdens.

Multilingual NLP standardizes reports and supporting documents into consistent formats regardless of their country of origin. Certifiers across different jurisdictions can review supplier information using a common digital template, reducing the need for repeated translations or clarifications. This standardization is especially valuable for exporters who must meet the requirements of multiple Halal authorities with distinct documentation rules [45].

NLP‐driven harmonization also improves the accuracy of cross‐border submissions by detecting semantic inconsistencies in technical documents, automatically identifying missing attestations, and ensuring that ingredient lists comply with regional religious interpretations [83].

ML models prioritize high‐risk suppliers, ingredients, or production lines based on historical compliance data and real‐time sensor readings. Auditors can then focus attention on the most critical cases instead of allocating equal effort to low‐risk facilities, improving both efficiency and the reliability of final approvals. Risk‐based audit scheduling represents a major advancement over fixed‐calendar audit cycles. AI can evaluate complex variables, including geographic sourcing risk, halal integrity vulnerability scores, supplier performance history, and anomaly clusters, to create dynamic audit priorities tailored to each facility. This approach increases audit precision while significantly reducing operational overhead for certification bodies.

By reducing manual labor and enabling smarter allocation of resources, AI lowers the overall cost of Halal certification. These savings make compliance more attainable for SMEs that may lack the financial capacity to undergo lengthy audits, allowing more producers to enter Halal markets, expand product diversity, and promote inclusive market growth. AI‐enabled self‐assessment dashboards also allow SMEs to prescreen their compliance status before formal audits, reducing the likelihood of nonconformities and enabling smoother certification cycles [90]. Cloud‐based platforms can provide step‐by‐step compliance guidance, automatically generate missing documentation, and offer real‐time alerts when production data deviate from Halal requirements, empowering SMEs with the same level of oversight typically available only to large multinational corporations.

Through automation, standardization, and intelligent risk management, AI streamlines Halal certification without sacrificing rigor. Certifying bodies can manage rising global demand more effectively, maintain consistent oversight across international supply chains, and ensure equitable access to certification and trade opportunities for producers of all sizes [36].

As global Halal supply chains continue to expand, scalable AI‐supported systems will become essential for harmonizing certification workflows, reducing administrative congestion, and creating a more inclusive, technology‐enabled Halal assurance ecosystem.

### 8.4. Strategic Importance for Halal Certification

While AI applications are valuable across many food certification schemes, the stakes are uniquely high for Halal systems. Religious adherence is absolute, and even a single prohibited substance can render an entire product haram, leading to recalls, reputational damage, and loss of consumer trust. At the same time, the global Halal market is projected to exceed USD 2 trillion in the coming decade, magnifying both commercial opportunities and the risks associated with certification failures [91]. These pressures highlight a critical need for technologies that ensure flawless compliance, provide real‐time verifiability, and support cross‐border harmonization requirements uniquely aligned with the technical strengths of AI‐driven systems.

AI technologies address these demands through three interrelated advantages that align closely with the requirements of Halal compliance.

#### 8.4.1. Precision

AI‐driven tools detect minute quantities of prohibited substances and identify subtle process deviations that might escape human inspection. ML algorithms analyze complex datasets to spot anomalies in supplier documentation, production parameters, or ingredient profiles. CV and AI‐enhanced spectroscopy can reveal traces of non‐Halal ingredients or improper slaughter practices with exceptional accuracy, ensuring that every stage of production meets religious standards [72].

Beyond technical detection, AI enhances interpretive precision by continuously updating risk models as new rulings, fatwa interpretations, or regulatory changes emerge. Adaptive systems allow certifiers to incorporate evolving religious interpretations, such as thresholds for incidental ethanol or acceptability of stunning methods, into automated compliance algorithms, reducing the likelihood of jurisprudential inconsistencies. This alignment of technical precision with jurisprudential exactness positions AI as a uniquely suitable tool for Halal systems, where compliance is both scientifically measurable and religiously mandated.

Customization for regional Halal practices is achieved through modular rule engines embedded within the AI system. These modules encode certifier‐specific requirements, regional jurisprudential interpretations, and nationally mandated thresholds, allowing AI outputs to reflect local religious expectations while operating on a shared technical infrastructure. This design enables culturally sensitive compliance assessment without fragmenting the underlying data architecture or analytical models [62].

#### 8.4.2. Trust

Blockchain integration and real‐time data analysis provide tamper‐proof, transparent records accessible to regulators, certifiers, retailers, and consumers. Each transaction and production step is stored in an immutable ledger, creating a verifiable chain of custody from farm to finished product. This transparency strengthens consumer confidence, reassures religious authorities, and facilitates international trade by making compliance evidence readily available across jurisdictions [60]. AI further strengthens trust by ensuring that records are not only traceable but also semantically verified. NLP systems validate the authenticity and consistency of digital certificates, while anomaly‐detection models flag irregularities that may indicate document tampering, unauthorized logo use, or hidden supplier substitutions. For religious authorities, AI‐generated audit trails offer high resolution, objective evidence that supports more confident issuance of fatwas or certification approvals. For consumers, especially younger digital‐native Muslim populations, transparent data builds confidence in Halal brands and counters skepticism fueled by frequent fraud reports.

#### 8.4.3. Scalability

AI enables certification bodies to manage expanding supply chains without overwhelming administrative workloads. Automated document processing, predictive risk assessment, and continuous monitoring allow certifiers to oversee a greater number of facilities and suppliers while maintaining high standards of accuracy. This scalability is essential as demand for Halal‐certified products continues to rise in both Muslim‐majority and non‐Muslim markets [86].

AI‐driven orchestration platforms allow certification bodies to consolidate global operations, coordinate multicountry audits, and synchronize data across diverse regulatory contexts. This is especially critical because Halal markets depend on extensive imports: Southeast Asia relies on global protein imports; the Gulf Cooperation Council (GCC) relies heavily on dairy and processed foods from Europe; and North America supplies Halal‐certified ingredients to dozens of countries.

As global food systems grow more interconnected, scalable AI systems ensure Halal integrity is maintained consistently, regardless of supply chain complexity or geographic distance.

#### 8.4.4. AI as a Model for Other Ethical and Religious Certification Systems

Through these combined capabilities, AI not only protects the integrity of Halal certification but also offers a model for other religious or ethical programs, including Kosher, Organic, Fair Trade, and sustainability labels where consumer confidence depends on verifiable compliance.

Several of these systems face challenges similar to Halal: complex supply chains, fraudulent labeling, inconsistent standards, and rising demand for transparency. AI‐driven Halal frameworks therefore serve as a blueprint for globally scalable, ethically aligned certification models.

By uniting precision, transparency, and scalability, AI creates a globally recognized benchmark for trustworthy, technology‐driven food certification. Ultimately, AI positions Halal certification at the forefront of global food assurance innovation, reinforcing its religious integrity while enhancing trade competitiveness and consumer trust.

## 9. Case Study and Illustrative Scenarios

This section illustrates how AI can reshape Halal certification by presenting (i) a comparative pilot scenario contrasting conventional and AI‐driven workflows and (ii) a real‐world implementation in a major poultry‐processing environment. Together, these cases demonstrate the operational, regulatory, and theological implications of AI adoption in Halal certification. A comparative summary of manual versus AI‐enabled Halal certification workflows is presented in Table 3.

**TABLE 3:** Comparison of manual versus AI‐integrated Halal certification workflows.

Certification type	Representative examples	Governing or accrediting body	Primary focus areas	Representative references
Governmental certifications	USDA Organic, European Union PDO/PGI, Codex Alimentarius	National or intergovernmental agencies such as the USDA, EU Commission, FAO/WHO Codex Committee	Food safety, production and processing standards, pesticide and additive restrictions, origin authenticity, labeling accuracy, trade facilitation, and harmonization of import/export regulations	[10–12, 22]
Third‐party or voluntary certifications	ISO 22000 Food Safety Management System, Fair Trade Certification, Non‐GMO Project Verified, Rainforest Alliance	Independent nongovernmental organizations (NGOs), industry coalitions, and international standards bodies such as ISO and Fair Trade International	Ethical sourcing, sustainability, equitable labor practices, environmental impact reduction, supply‐chain transparency, and consumer trust through independent verification	[13, 20, 22]; [52]
Religious certifications	Halal (food and nonfood products), Kosher (meat and packaged goods), Hindu vegetarian labels	Religious authorities and accredited certifying bodies such as JAKIM (Malaysia), IFANCA (USA), MUI (Indonesia), and OU (Kosher Union)	Compliance with religious dietary law, ritual slaughter supervision, ingredient permissibility, cross‐contamination prevention, facility hygiene, and theological oversight ensuring spiritual and physical integrity	[9, 15–17, 29]

Within this framework, accuracy is achieved through multimodal verification, transparency through immutable and auditable data trails, and efficiency through automation and predictive analytics. These dimensions collectively define effective Halal certification in complex global supply chains.

### 9.1. Pilot Implementation Scenario: Manual vs. AI‐Integrated Workflow

A multinational snack manufacturer seeking Halal certification for a new extruded product relied on a traditional, manual workflow that required multiple layers of document review and on‐site verification. Supplier documentation including ingredient specifications, slaughter certificates, and processing records from more than fifty global suppliers had to be manually collected, reviewed, and translated. Human auditors cross‐referenced these documents against Halal ingredient databases, a process that typically required 3–4 weeks due to multinational sourcing, inconsistent terminology, and multilingual records.

After document review, certifiers conducted on‐site audits at several manufacturing locations to verify segregation of Halal and non‐Halal production lines, sanitation protocols, and compliance with slaughter requirements. Only after all data were verified and audits completed was certification granted, extending the overall timeline to eight to 10 weeks. Delays caused by incomplete documentation, inconsistent supplier formats, or auditor travel constraints often prolonged timelines and increased costs [19, 75].

Integrating AI dramatically streamlined this workflow. Automated optical character recognition and NLP extracted and translated ingredient lists, technical specifications, and supplier certificates within minutes, eliminating manual transcription errors. ML algorithms cross‐checked these records against accredited Halal ingredient registries, flagging high‐risk suppliers or ambiguous ingredients within hours. IoT sensors embedded in processing lines captured real‐time data on temperature, sanitation cycles, and equipment segregation, which AI models analyzed to verify compliance with Halal protocols continuously.

Every verification step was recorded on a blockchain ledger, generating a tamper‐proof, time‐stamped compliance trail accessible to certifiers, regulators, and downstream buyers. Continuous monitoring enabled the facility to maintain an “always‐certified” status, allowing certifiers to renew approval based on dynamic data rather than periodic inspections [40, 74].

With AI integration, total certification time was reduced from eight to 10 weeks to approximately 2–3 weeks. Audit costs decreased by 30%–40% due to targeted inspections and fewer site visits. The immutable blockchain record accelerated export approvals by providing regulators with instant access to verified compliance data. This scenario demonstrates how AI can transform Halal certification from a document‐heavy, reactive process into a continuous, data‐driven compliance model that protects religious integrity while promoting rapid market access [40, 92].

### 9.2. Industry Case Study: AI in Meat Processing

A leading poultry processor in Southeast Asia implemented an AI‐enabled Halal certification system between 2021 and 2023 across high‐throughput slaughter and processing facilities supplying both domestic and export markets under the oversight of a nationally recognized Halal certification authority. The objective was to strengthen compliance oversight while maintaining religiously mandated control over slaughter and processing.

The system integrated CV, NLP, and blockchain across multiple operational stages. High‐resolution cameras monitored knife placement, animal orientation, handling practices, and bleeding efficiency. Deep‐learning algorithms analyzed continuous video streams to ensure each step aligned with Halal slaughter guidelines. NLP systems translated and cross‐checked multilingual supplier documents, detecting ambiguous ingredient descriptions, nonstandard terminology, or incomplete certification records. Blockchain technology established an immutable chain of custody from animal intake to final packaging, ensuring traceability and preventing document tampering [7, 74].

Implementation progressed from planning to live operation within 6 months, encompassing system integration, staff training, and calibration of CV models to interpret local slaughter practices. Religious authorities played a central role throughout the process, verifying AI‐generated alerts and ensuring the technology adhered to theological expectations.

The pilot delivered substantial measurable gains. Noncompliance detection accuracy increased from 93% under manual inspection to 99.5% with AI‐assisted monitoring. Document review timelines were reduced from approximately fifteen days to less than forty‐eight hours, accelerating certification renewals and export approvals. Blockchain eliminated duplicate and counterfeit certificates by creating a unified, tamper‐proof record of all certification events [40].

By maintaining religious oversight of AI outputs, the project demonstrated both technological feasibility and cultural acceptability. The case illustrates how AI can enhance the scientific, operational, and scriptural reliability of Halal certification and provides a scalable model for broader global adoption.

### 9.3. Lessons Learned and Broader Implications

#### 9.3.1. Hybrid Oversight

AI enhances speed and accuracy but cannot replace human judgment in matters involving religious compliance, animal welfare, or theological interpretation. Islamic scholars, auditors, and certifiers retain final decision‐making authority, using AI‐generated evidence to support rulings. This hybrid model ensures that certification remains scientifically rigorous without compromising its spiritual legitimacy [93].

#### 9.3.2. Early Stakeholder Engagement

Successful AI adoption requires active collaboration among religious authorities, regulators, manufacturers, and technology developers from the earliest planning stages. This collaboration shapes acceptable monitoring practices, data‐sharing protocols, and privacy safeguards. Early engagement ensures that AI systems reflect regional jurisprudence and local market expectations [18].

#### 9.3.3. Scalability and Export Advantage

Continuous AI‐enabled certification reduces shipment lead times and accelerates access to premium export markets. Real‐time verification allows exporters to respond quickly to demand surges, schedule shipments efficiently, and avoid delays associated with periodic audits. This capability is particularly advantageous for perishable goods and complex multinational supply chains [74].

#### 9.3.4. Transferability to Other Certifications

AI‐driven verification frameworks developed for Halal certification can support other assurance systems that share challenges related to fraud, traceability, and compliance, such as Kosher, Organic, Fair Trade, allergen‐free, and sustainability certifications. These systems can leverage AI‐powered document analysis, blockchain‐based traceability, and real‐time monitoring for more reliable and efficient verification [92].

Collectively, these lessons show that combining advanced technology with human oversight and cross‐sector collaboration creates certification systems that are accurate, transparent, and scalable across diverse food‐assurance contexts.

### 9.4. Toward Industry‐Wide Adoption

Pilot programs demonstrate that AI can shift Halal certification from periodic inspection toward a continuous, predictive compliance model. Scaling these pilots into industry‐wide implementation will require coordinated regulatory, technological, and theological initiatives.

#### 9.4.1. Shared Data Standards

Global harmonization of data formats and system architectures is essential for cross‐border recognition of AI‐verified records. Standardized frameworks for ingredient databases, audit modules, and blockchain entries would allow mutual recognition of digital compliance information across jurisdictions, reducing redundancy and simplifying export approvals [70].

#### 9.4.2. Regulatory Harmonization

To avoid inconsistencies in interpretation among Islamic schools of thought, AI‐assisted certification protocols must be aligned with religious authorities at national and international levels. Collaborative efforts among Halal accreditation bodies, religious scholars, and government agencies can define baseline requirements for AI‐enabled monitoring and establish mutual recognition agreements that support seamless trade [94].

#### 9.4.3. Capacity Building

Auditors, certifiers, and plant personnel require training in AI system interpretation, data validation, and blockchain traceability. Educational programs that combine Halal jurisprudence with data analytics and digital audit methods will ensure that experts can exercise informed oversight rather than relying on AI as a “black box” [75].

#### 9.4.4. Privacy and Security Safeguards

Effective data governance policies must protect confidential commercial information and ensure ethical use of AI and blockchain. Encryption, access controls, and transparent consent mechanisms supported by third‐party cybersecurity audits are essential for maintaining trust among producers, certifiers, regulators, and consumers [93].

Addressing these priorities will enable the global food industry to scale AI‐enabled Halal certification systems that are faster, more reliable, and more trustworthy, while establishing a model for modernizing assurance frameworks across other food sectors.

## 10. Future Perspectives

The integration of AI into Halal certification remains at an early stage, yet emerging evidence indicates that it will play a transformative role in modernizing food‐assurance systems worldwide. As global supply chains expand in complexity and Halal markets continue to grow, the next decade will likely see AI shift from localized pilot deployments to a foundational component of certification infrastructure. Achieving this transition will require coordinated progress across regulatory, ethical, technological, and institutional domains, supported by strong collaboration among religious authorities, certifiers, industry stakeholders, and government agencies.

### 10.1. Policy and Regulatory Directions

#### 10.1.1. Global Harmonization of Standards

Halal certification practices vary significantly across jurisdictions due to differences in Islamic jurisprudence, national regulations, and accreditation systems. For AI‐enabled certification to be recognized across borders, internationally aligned data standards and audit protocols are needed. These include standardized blockchain structures, common metadata formats for digital certificates, interoperable ingredient databases, and shared calibration guidelines for sensor‐based monitoring. Regional bodies such as the Organization of Islamic Cooperation (OIC) and global leaders like JAKIM (Malaysia), IFANCA (United States), and MUI (Indonesia) are well positioned to coordinate harmonization efforts and establish mutual recognition agreements. Such frameworks would reduce redundancy, prevent conflicting rulings, and streamline export approvals by ensuring that digital compliance records generated in one country remain valid in others.

#### 10.1.2. Regulatory Oversight of AI

The adoption of AI in Halal certification requires clear regulatory controls to protect religious integrity, commercial confidentiality, and consumer trust. Priority areas include transparency requirements for AI model decision logic, data‐governance safeguards for shared records, and liability protocols addressing outcomes where AI‐generated alerts conflict with human assessments. Government agencies and religious bodies must jointly define conditions under which AI outputs are admissible as compliance evidence, including accuracy thresholds, model‐validation procedures, and audit trails. By establishing explicit rules for responsibility, oversight, and dispute resolution, regulators can provide a stable foundation for wider uptake of AI technologies in faith‐sensitive markets.

### 10.2. Ethical and Social Considerations

#### 10.2.1. Preserving Religious Authority

While AI can provide unprecedented analytical depth, final certification authority must remain with qualified religious scholars and accredited Halal certifiers. AI should serve as an evidence‐generation tool that enhances, rather than replaces, theological decision‐making. Clear governance policies must delineate the respective roles of AI systems and human certifiers to preserve the sanctity of religious rulings, especially in borderline or context‐dependent scenarios involving slaughter practices, processing aids, or region‐specific jurisprudential interpretations.

#### 10.2.2. Mitigating Algorithmic Bias

ML systems may inadvertently introduce biases if trained on unrepresentative datasets or narrow regional practices. In the Halal context, this could result in over‐flagging certain suppliers, misinterpreting culturally specific practices, or under‐detecting rare forms of adulteration. Regular algorithm audits, diverse training datasets, and transparent model documentation are essential to ensure fairness and accuracy. Engagement of Halal scholars in reviewing AI model criteria further reduces the risk of misalignment with religious expectations.

#### 10.2.3. Transparency to Consumers

Consumers increasingly expect visibility into certification processes, particularly in religious or ethical markets. AI, combined with blockchain traceability, can support consumer‐facing platforms that provide structured summaries of a product’s verification history through QR codes or mobile applications. These systems must balance transparency with data privacy, providing key compliance milestones without exposing sensitive commercial information. Enhanced visibility can strengthen consumer trust, particularly among younger and non‐Muslim demographics who associate Halal certification with hygiene, traceability, and ethical sourcing.

### 10.3. Technological Opportunities

#### 10.3.1. AI‐Blockchain Synergy

The integration of blockchain’s immutable recordkeeping with AI’s analytical capabilities offers one of the strongest safeguards against fraud and data manipulation. AI can continuously scan blockchain entries to detect patterns such as missing timestamps, improbable transport sequences, or inconsistent supplier records. Future systems may allow automated cross‐border validation of Halal certificates, reducing manual verification and eliminating counterfeit documentation. This synergy provides the foundation for a shared global Halal compliance ledger recognized by multiple regulatory and religious authorities.

#### 10.3.2. Real‐Time Predictive Certification

As IoT sensors evolve and AI models gain sophistication, Halal certification can transition from static, periodic audits to dynamic, predictive oversight. AI can anticipate potential noncompliance such as increased cross‐contamination risk or emerging supplier vulnerabilities before they materialize. This predictive capability supports early corrective action, reducing recalls, strengthening preventive control systems, and enabling facilities to maintain an “always‐certified” operational status. This shift aligns with broader food‐safety modernization trends and enhances Halal certification’s competitiveness.

#### 10.3.3. Integration With Emerging Technologies

New scientific tools offer additional pathways to strengthen Halal verification. Digital twins can model facility operations, allowing virtual audits and stress‐testing of segregation protocols before physical changes occur. Satellite imaging supports remote verification of livestock, agricultural sourcing, and land‐use compliance. Advances in DNA nanotechnology and portable molecular diagnostics promise faster and more sensitive detection of species‐specific contaminants. AI can fuse these datasets—physical, digital, environmental, and molecular—into unified risk models, enabling earlier detection of adulteration or supply‐chain anomalies.

### 10.4. Roadmap for Industry Adoption

Long‐term relevance of AI models is maintained through structured lifecycle management, including periodic retraining with updated datasets, validation against benchmark compliance cases, and incorporation of new regulatory or jurisprudential guidance. Explainable AI tools enable auditors and certifiers to review model logic and outputs, preventing performance degradation or unintended bias. Human‐in‐the‐loop governance ensures that updates enhance, rather than override, expert judgment [79].

The proposed system is designed to adapt to future technological advancements and regulatory changes through modular architecture and periodic model updates. As new analytical tools, regulatory requirements, or Halal standards emerge, AI components can be recalibrated or retrained without restructuring the entire certification framework. Version control, audit logs, and backward compatibility mechanisms ensure continuity and traceability of compliance decisions over time [95].

#### 10.4.1. Capacity Building

Training is essential for auditors, plant operators, and religious scholars to interpret AI outputs and integrate them into existing workflows. Curricula should include AI fundamentals, digital auditing methods, blockchain literacy, and data‐governance principles, ensuring that stakeholders understand how to interpret algorithmic evidence without becoming overly dependent on automation.

#### 10.4.2. Collaborative Governance

Multistakeholder governance frameworks involving certifiers, technology providers, regulatory authorities, religious scholars, and consumer groups can jointly establish best practices and ethical guidelines. These collaborative structures are critical for ensuring that systems meet both theological expectations and international technical standards, especially in settings where multiple schools of jurisprudence intersect with diverse national regulatory regimes.

#### 10.4.3. Scalable Infrastructure

Large‐scale adoption requires secure, interoperable digital infrastructure, including cloud‐based data repositories, tamper‐proof blockchain ledgers, standardized interfaces for IoT devices, and cross‐border data‐sharing agreements. A unified infrastructure lowers costs, avoids duplication, and ensures system reliability across diverse markets.

#### 10.4.4. Incremental Deployment

A pragmatic rollout strategy begins with high‐risk or high‐value areas such as slaughter verification, gelatin authentication, or flavoring‐alcohol detection, where AI provides immediate value. Early wins in these focused applications build trust among certifiers and scholars, encourage industry adoption, and create a financial case for broader deployment across full supply chains.

### 10.5. Broader Implications

Although this review focuses on Halal certification, the frameworks and insights apply equally to Kosher, Organic, Fair Trade, allergen‐free, sustainability, and other assurance systems. All face parallel challenges: complex global supply chains, rising consumer expectations for transparency, and the need to maintain integrity across multiple regulatory environments.

AI‐powered systems, ML, NLP, CV, spectroscopy, DNA authentication, and blockchain can address these challenges by enabling more accurate fraud detection, more efficient document verification, and more robust traceability. The Halal sector provides a particularly compelling test case because religious adherence requires absolute precision; successful AI integration here demonstrates that advanced technologies can uphold, and even strengthen, ethical and religious principles rather than replacing or diminishing them.

By showing that AI can augment human expertise while safeguarding theological integrity, Halal certification offers a globally relevant model for modernizing food‐assurance systems. As food systems become more digitalized, the Halal sector’s experience with AI adoption may guide other certification bodies toward more transparent, efficient, and trustworthy compliance frameworks.

## 11. Conclusion

AI will not supplant the human judgment, religious authority, or ethical responsibility that form the foundation of Halal certification; however, it is positioned to become a critical partner in preserving these core values. As global supply chains expand and product formulations grow more complex, the next decade is likely to witness a transition from periodic, paper‐based audits toward continuous, predictive, and tamper‐resistant verification systems. AI’s capacity to analyze large, heterogeneous datasets, detect subtle irregularities, and provide uninterrupted oversight offers precision and responsiveness far beyond what traditional methods can achieve alone.

This transformation extends beyond technological capability. It requires robust governance frameworks, clearly articulated regulatory pathways, and sustained collaboration among religious scholars, certification bodies, technology developers, food manufacturers, and policymakers. Standard setting will play a pivotal role in ensuring that AI tools remain aligned with diverse interpretations of Islamic jurisprudence while supporting interoperability across countries, accreditation systems, and supply‐chain infrastructures. The success of AI‐enabled certification will also depend on investments in human capital, including training auditors, plant operators, and certification officers to interpret AI outputs, evaluate algorithmic evidence, and integrate digital findings into established decision‐making processes without diminishing theological oversight.

When effectively implemented, AI‐supported Halal certification can deliver benefits far beyond operational efficiency. It can reinforce religious integrity by providing objective, high‐resolution compliance data; enhance consumer trust through transparent, tamper‐proof verification; and strengthen global market competitiveness by shortening certification timelines and improving traceability. Furthermore, the Halal sector’s experience offers a broader template for technology‐driven assurance systems. The same principles, hybrid oversight, data integrity, algorithmic transparency, and cross‐border harmonization are applicable to Kosher, Organic, Fair Trade, sustainability, allergen‐free, and other value‐based certifications.

By integrating human stewardship with intelligent automation, AI has the potential to redefine food certification as a continuous, transparent, and universally trusted process. In doing so, it demonstrates that technological innovation and faith‐based principles are not opposing forces but mutually reinforcing pillars capable of supporting a safer, more ethical, and more globally coherent food system.

**Nomenclature**
AI: Artificial intelligence
ANN: Artificial neural network
API: Application programming interface
CV: Computer vision
DNA‐PCR: DNA polymerase chain reaction
DL: Deep learning
FTIR: Fourier‐transform infrared spectroscopy
ICT: Information and communication technologies
IoT: Internet of Things
ISO: International organization for standardization
ML: Machine learning
NLP: Natural language processing
OCR: Optical character recognition
PDO: Protected Designation of Origin
PGI: Protected geographical indication
RFID: Radio‐frequency identification
SME: Small and medium‐sized enterprise
SVM: Support vector machine
XAI: Explainable artificial intelligence

## Author Contributions

Mian N. Riaz: conceptualization, resources, review and editing, and supervision.

Fariha Irshad: writing, reviewing, and editing; methodology; and investigation.

Muhammad Bilal Haider: reviewing and editing.

## Funding

No funding was received for this manuscript.

## Conflicts of Interest

The authors declare no conflicts of interest.

## Data Availability

Data sharing is not applicable to this article, as no datasets were generated or analyzed during the current study.
